# Examination of the Accuracy of Existing Overdose Surveillance Systems

**DOI:** 10.1001/jamanetworkopen.2023.20789

**Published:** 2023-06-28

**Authors:** Jennifer Griffith, Laura C. Chambers, Benjamin D. Hallowell, Ashley Gaipo, Craig Mailloux, Janette Baird, Francesca L. Beaudoin, Elizabeth A. Samuels

**Affiliations:** 1 Department of Epidemiology, Brown University School of Public Health, Providence, Rhode Island; 2 Substance Use Epidemiology Program, Rhode Island Department of Health, Providence; 3 Department of Emergency Medicine, Alpert Medical School of Brown University, Providence, Rhode Island; 4 Data Analytics and Insights, Lifespan Corporate Services, Providence, Rhode Island; 5 Overdose Prevention Program, Rhode Island Department of Health, Providence; 6 Department of Emergency Medicine, University of California, Los Angeles

## Abstract

**Importance:**

Health departments have used a variety of methods for overdose surveillance, and the Centers for Disease Control and Prevention (CDC) is implementing a standardized case definition to improve overdose surveillance nationally. The comparative accuracy of the CDC opioid overdose case definition vs existing state opioid overdose surveillance systems is unknown.

**Objective:**

To evaluate the accuracy of the CDC opioid overdose case definition and existing Rhode Island Department of Health (RIDOH) state opioid overdose surveillance system.

**Design, Setting, and Participants:**

This cross-sectional study of ED opioid overdose visits was conducted at 2 EDs in Providence, Rhode Island, at the state’s largest health system from January to May 2021. Electronic health records (EHRs) were reviewed for opioid overdoses identified by the CDC case definition and opioid overdoses reported to the RIDOH state surveillance system. Included patients were those at study EDs whose visit met the CDC case definition, was reported to the state surveillance system, or both. True overdose cases were confirmed by EHR review using a standard case definition; 61 of 460 EHRs (13.3%) were double reviewed to estimate classification accuracy. Data were analyzed from January through May 2021.

**Main Outcome and Measure:**

Accurate identification of an opioid overdose was assessed by estimating the positive predictive value of the CDC case definition and state surveillance system using results from the EHR review.

**Results:**

Among 460 ED visits that met the CDC opioid overdose case definition, were reported to the RIDOH opioid overdose surveillance system, or both (mean [SD] age, 39.7 [13.5] years; 313 males [68.0%]; 61 Black [13.3%], 308 White [67.0%], and 91 other race [19.8%]; and 97 Hispanic or Latinx [21.1%] among each patient visit), 359 visits (78.0%) were true opioid overdoses. For these visits, the CDC case definition and RIDOH surveillance system agreed that 169 visits (36.7%) were opioid overdoses. Of 318 visits meeting the CDC opioid overdose case definition, 289 visits (90.8%; 95% CI, 87.2%-93.8%) were true opioid overdoses. Of 311 visits reported to the RIDOH surveillance system, 235 visits (75.6%; 95% CI, 70.4%-80.2%) were true opioid overdoses.

**Conclusions and Relevance:**

This cross-sectional study found that the CDC opioid overdose case definition more often identified true opioid overdoses compared with the Rhode Island overdose surveillance system. This finding suggests that using the CDC case definition for opioid overdose surveillance may be associated with improved data efficiency and uniformity.

## Key Points

**Question:**

Does the Centers for Disease Control and Prevention (CDC) opioid overdose case definition or Rhode Island state surveillance system more often identify true emergency department visits for opioid overdose?

**Findings:**

This cross-sectional study of 460 emergency department visits identified as opioid overdoses by either or both approaches found that the CDC opioid overdose case definition more accurately identified true emergency department visits for opioid overdoses than the state surveillance system.

**Meaning:**

This finding suggests that it may be advantageous to use the CDC case definition, which is available for use by states through the CDC Electronic Surveillance System for the Early Notification of Community-Based Epidemics, for opioid overdose surveillance in Rhode Island.

## Teaser

This cross-sectional study investigates the accuracy of the Centers for Disease Control and Prevention opioid overdose case definition compared with a state surveillance system.

## Introduction

Opioid overdose surveillance is a key public health strategy to inform rapid and targeted responses to the overdose crisis in the US.^1,2^ States have historically maintained their own surveillance systems, which vary in data collection methods and content and case definitions. The US Centers for Disease Control and Prevention (CDC) has been leading a national effort to standardize opioid overdose syndromic surveillance using emergency department (ED) visit data and a standard opioid overdose case definition from electronic health record (EHR) chief complaint field and opioid overdose–related diagnostic code data.^3,4^ However, the relative performance of the CDC opioid overdose case definition vs existing state-based systems is unknown. We assessed the accuracy of the CDC opioid overdose case definition and Rhode Island overdose surveillance system in identifying opioid overdose ED visits by comparing them with a reference based on EHR review.

## Methods

This retrospective cross-sectional study was conducted in accordance with the Strengthening the Reporting of Observational Studies in Epidemiology (STROBE) reporting guideline. The Lifespan Health System Institutional Review Board approved this study.

### Study Sample and Data

We conducted a cross-sectional study of opioid overdose ED visits at 2 large urban EDs in Providence, Rhode Island, from January 1 through May 31, 2021. We reviewed EHR data for opioid overdoses for adult patients identified by the CDC opioid overdose case definition or reported to the RIDOH state surveillance system. Cases were included in the study if they were captured by either surveillance system during the 5-month study period.

### CDC Opioid Overdose Case Definition

The CDC opioid overdose case definition uses information from EHR chief complaint fields, opioid overdose–related diagnostic codes (*International Classification of Diseases, Ninth Revision* [*ICD-9*] and *International Statistical Classification of Diseases and Related Health Problems, Tenth Revision* [*ICD-10*]), and specific SNOMED CT codes (eFigure in Supplement 1).^3^ Hospitals automatically send ED EHR data to RIDOH in near real time, and RIDOH applies the CDC case definition for CDC reporting and inclusion in the CDC Electronic Surveillance System for the Early Notification of Community-Based Epidemics (ESSENCE).^4^ This process was under development at the time of this study. For this study, we identified ED visits meeting the CDC opioid overdose case definition through electronic query of the EHR.

### Rhode Island State Surveillance System

Since 2014, Rhode Island has required EDs to report suspected opioid overdoses to RIDOH within 48 hours through a secure, web-based platform.^5^ To identify records for reporting, a daily list of potential opioid overdoses is generated by EHR query for visits with a naloxone order, an opioid- or overdose-related chief complaint, or an opioid overdose–related discharge diagnosis. A quality assurance nurse manually reviews identified records to determine whether the visit was an opioid overdose.^5^ Subsequently, a quality assurance nurse or unit secretary manually enters deidentified, individual-level data into an online reporting form. For this study, we identified ED visits reported to the state surveillance system during the study period using a database of reported records maintained by the hospital quality and patient safety division.

### EHR Review

We conducted a manual EHR review of cases identified by the CDC opioid overdose case definition, reported to the RIDOH surveillance system, or both to determine whether these identified visits were true opioid overdoses using a standard case definition. We defined a true opioid overdose as an event in which a patient developed respiratory depression, loss of consciousness, or both immediately after the patient had reportedly ingested an opioid or naloxone was administered with improvement in respiration or consciousness. EHR fields reviewed included emergency medical services run sheets, lab results, orders, and EHR documentation by clinicians, nurses, and social and community health workers.

EHRs were reviewed by 2 data extractors (J.G. and A.G.) trained and supervised by a senior study team member and emergency physician (E.A.S.). Patient sociodemographic data, including race and ethnicity, collected for the study were from EHR data, which is a combination of hospital registration–recorded and patient self-reported data. Race, ethnicity, and sex categories available in the EHR were, respectively, American Indian or Alaskan Native, Asian, Black or African American, Hawaiian or other Pacific Islander, White, not documented, and other; Hispanic or Latinx, not Hispanic or Latinx, not documented, and other; and female, male, and other. For analysis, race and ethnicity categories were grouped into Black, White, and other and Hispanic or Latinx, not Hispanic or Latinx, and unknown, respectively, due to small sample sizes. Most individuals in the other racial category identified as Hispanic or Latino. We assessed age, sex, race, and ethnicity to describe the demographic composition of the study population overall and in study groups. Data extractors entered ED visit data (patient sociodemographic, prehospital, and ED visit characteristics) into a research electronic data capture^6^ form and determined whether the ED visit met the standard case definition. A subsample of EHRs (61 of 460 EHRs [13.3%]) were double reviewed (E.A.S.) for accuracy and to determine interrater reliability.

### Statistical Analysis

Study data were analyzed using Stata statistical software version 17 (StataCorp) and RStudio statistical software version 4.1.2 (RStudio). We descriptively compared agreement between the CDC case definition and state surveillance system. We separately estimated the positive predictive value (PPV) and exact binomial 95% CI^7,8^ for each approach compared with the standard case definition (ie, percentage of visits classified as opioid overdoses that were truly opioid overdoses) and descriptively compared 95% CIs. We calculated a κ score to estimate the interrater reliability of each data extractor compared with the emergency medicine physician for our standard case definition. The level of statistical significance was defined a priori as α = .05, and statistical tests were 2-sided. Data were analyzed from January through May 2021.

## Results

Between January 1, 2021, and May 31, 2021, there were 460 ED visits (mean [SD] age, 39.7 [13.5] years; 313 males [68.0%]; 61 Black [13.3%], 308 White [67.0%], and 91 other race [19.8%]; and 97 Hispanic or Latinx [21.1%] among each patient visit) meeting the CDC opioid overdose case definition (318 visits), reported to the Rhode Island state surveillance system (311 visits), or both at study EDs (Table 1). Of 460 ED visits, 359 visits (78.0%) were confirmed as true opioid overdoses by EHR review. Among ED visits classified as opioid overdoses by either approach, the approaches agreed for 169 visits (36.7%) (Table 2). Patient demographics were similar between reporting approaches, but a lower proportion of visits reported to the state system had prehospital naloxone administration (208 visits [66.9%] vs 277 visits [87.1%]) or mention of opioid overdose in nursing (218 visits [70.1%] vs 286 visits [89.9%]) or clinician (241 visits [77.5%] vs 296 [93.1%]) notes compared with the CDC case definition (Table 1).

**Table 1.** Characteristics of ED Visits Classified as Opioid Overdoses

Characteristic	ED visits, No. (%) (N = 460)		
	Identified by CDC case definition (n = 318)	Reported to state surveillance system (n = 311)	True opioid overdoses identified by either approach (n = 359)
**Patient**			
Age, mean (SD), y	39.4 (12.8)	38.8 (13.4)	38.5 (12.7)
Sex^a^			
Female	90 (28.3)	101 (32.5)	106 (29.5)
Male	227 (71.4)	210 (67.5)	252 (70.2)
Other	<10^b^	<10^b^	<10^b^
Race^a^			
Black or African American	37 (11.6)	45 (14.5)	48 (13.4)
White	206 (64.8)	204 (65.6)	231 (64.3)
Other	75 (23.6)	62 (20.0)	80 (22.4)
Hispanic or Latino ethnicity^a^			
Yes	79 (24.8)	65 (20.9)	83 (23.1)
No	232 (73.0)	239 (76.8)	268 (74.7)
Unknown	<10^b^	<10^b^	<10^b^
Naloxone administered prior to ED presentation			
Yes	277 (87.1)	208 (66.9)	311 (86.6)
No	41 (12.9)	103 (33.2)	48 (13.3)
**ED visit**			
Site			
Hospital A	212 (66.7)	192 (61.7)	233 (64.9)
Hospital B	106 (33.3)	119 (38.3)	126 (35.1)
Mention of opioid overdose^c^			
EMS run sheet	90 (28.3)	62 (19.9)	101 (28.1)
Nursing note	286 (89.9)	218 (70.1)	324 (90.3)
Clinician note	296 (93.1)	241 (77.5)	346 (96.4)
Primary discharge diagnosis			
Opioid use, abuse, or dependence with intoxication	37 (11.6)	33 (10.6)	35 (9.7)
Poisoning	241 (75.8)	210 (67.5)	285 (79.4)
Other	40 (12.6)	68 (21.9)	39 (10.9)
Patient disposition			
Admitted	50 (15.7)	78 (25.1)	58 (16.2)
Discharged	214 (67.3)	190 (61.1)	245 (68.2)
Other^c^	54 (17.0)	43 (13.8)	56 (15.6)
Select services^d^			
Naloxone administered	60 (18.9)	50 (16.1)	70 (19.5)
Urine drug screen completed	149 (46.9)	148 (47.6)	178 (49.6)
Positive for opioids^e^	145 (97.3)	137 (92.6)	174 (97.8)
Buprenorphine administered	11 (3.5)	<10^b^	11 (3.1)
Buprenorphine prescription provided	<10^b^	<10^b^	<10^b^
Behavioral counseling provided	122 (38.4)	109 (35.0)	144 (40.1)
Take-home naloxone kit ordered	193 (60.7)	166 (53.4)	235 (65.5)
Referral to recovery center provided	113 (81.9)	77 (72.6)	129 (81.6)

Abbreviations: CDC, Centers for Disease Control and Prevention; ED, emergency department; EMS, emergency medical services.

^a^ Race, ethnicity, and sex categories were combined into listed categories due to low counts and data categories available within the electronic health record. Race, ethnicity, and sex categories in the electronic health record were American Indian or Alaskan Native, Asian, Black or African American, Hawaiian or other Pacific Islander, White, not documented, and other; Hispanic or Latinx, not Hispanic or Latinx, not documented, and other; and female, male, and other, respectively.

^b^ Values less than 10 suppressed to protect patient privacy.

^c^ Other patient disposition categories included against medical advice, eloped, expired, left without being seen, and transfer.

^d^ Not mutually exclusive categories.

^e^ Percentages are among 149 visits at which a urine drug screen for opioids was completed.

**Table 2.** Agreement Between Approaches in Opioid Overdose Classification

Identified by CDC case definition	Reported to state surveillance system		
	Yes	No	Total
Yes	169	149	318
No	142	NA^a^	142
Total	311	149	460

Abbreviations: CDC, Centers for Disease Control and Prevention; NA, not applicable.

^a^ Not assessed given that all records in the study sample were identified by CDC case definition or reported to the state surveillance system.

EHR review interrater reliability was high (data extractor 1: κ = 0.94; 95% CI, 0.84-1.00; data extractor 2: κ = 1.00; 95% CI, 1.0-1.0) (eTable in Supplement 1). Of 318 visits meeting the CDC opioid overdose case definition, 289 visits (90.9%) were true opioid overdoses (PPV = 90.8%; 95% CI, 87.2%-93.8%) (Table 3). Of 311 visits reported to the state surveillance system, 235 visits (75.6%) were true opioid overdoses (PPV = 75.6%; 95% CI, 70.4%-80.2%).

**Table 3.** Performance of Classification Approaches vs Standard Case Definition

Classification	Met true opioid overdose case definition		
	Yes	No	Total
**Identified by CDC case definition**			
Yes^a^	289	29	318
No	70	72	142
Total	359	101	460
**Reported to state surveillance system**			
Yes^b^	235	76	311
No	124	25	149
Total	359	101	460

Abbreviation: CDC, Centers for Disease Control and Prevention.

^a^ The positive predictive value was 90.8% (95% CI, 87.2%-93.8%).

^b^ The positive predictive value was 75.6% (95% CI, 70.4%-80.2%).

## Discussion

In this cross-sectional study of ED opioid overdose visits, we found that the CDC opioid overdose case definition more often identified true opioid overdoses than the state surveillance system (90.8% vs 75.6%). RIDOH uses state surveillance data to inform rapid and targeted overdose responses.^9,10^ Our findings suggest that transitioning to the CDC opioid overdose case definition through ESSENCE may be associated with improved surveillance accuracy and timeliness and a decrease in the reporting burden for hospital staff of manual reporting, which is time and labor intensive, subject to human error, and often delayed. Given limited resources for overdose prevention, it may be preferable for overdose surveillance systems to identify true opioid overdoses rather than a mixture of true opioid overdoses and other visit types, so long as it does not significantly undercount overdoses and specific subgroups of patients are not systematically underrepresented. Many departments of health and community harm reduction organizations are working with limited resources; to effectively reduce overdose deaths, resources should be directed where they are needed most, in areas with confirmed high overdose incidence. Further studies are needed to evaluate the sensitivity of this case definition on its own and in comparison with other overdose surveillance approaches to ensure generalizability and accurate account of overdose incidence for allocation of overdose prevention resources.

### Limitations

This study has several limitations. As a retrospective EHR review, this study was limited by reporting and recording bias in the EHR which may result in inaccurate classification of ED visits as opioid overdoses and potentially undercounting of overdose events. A major limitation of this study was that our approach may have missed some true opioid overdoses given that it was not feasible to manually review all ED records during the study period. Consequently, we were not able to measure other aspects of the CDC case definition performance (eg, sensitivity or specificity). While the CDC case definition does not erroneously classify many other types of visits as opioid overdoses, the number of true opioid overdose visits that it misses is uncertain.

## Conclusions

This cross-sectional study found that the CDC opioid overdose case definition more often identified true opioid overdoses than the existing Rhode Island state surveillance system. This finding suggests that an automated process through ESSENCE may be associated with improved timeliness and uniformity across hospitals, health systems, and states, which is crucial for collection of actionable surveillance data to inform community response efforts.

## Notes

Supplement 1. **eTable.** Interrater Reliability for Classifications of True Opioid Overdose for 2 Data Extractors vs Emergency Medicine Physician **eFigure.** Centers for Disease Control and Prevention Drug Overdose Surveillance and Epidemiology System Chief Complaint and Discharge Diagnosis Search Terms for All Suspected Opioid Overdose

Supplement 2.
Data Sharing Statement
