# Supplementary material for: Examination of the Accuracy of Existing Overdose Surveillance Systems
Source: JAMA Netw Open. 2023 Jun 28;6(6):e2320789. doi: 10.1001/jamanetworkopen.2023.20789 (PMC10308251; doi:10.1001/jamanetworkopen.2023.20789)
Supplement: Supplement 1. — eTable. Interrater Reliability for Classifications of True Opioid Overdose for 2 Data Extractors vs Emergency Medicine Physician eFigure. Centers for Disease Control and Prevention Drug Overdose Surveillance and Epidemiology System Chief Complaint and Discharge Diagnosis Search Terms for All Suspected Opioid Overdose [file jamanetwopen-e2320789-s001.pdf]

Supplemental Online Content

Griffith J, Chambers LC, Hallowell BD, et al. Examination of the accuracy of existing overdose surveillance systems. *JAMA Netw Open.* 2023;6(6):e2320789.
doi:10.1001/jamanetworkopen.2023.20789

eTable. Interrater Reliability for Classifications of True Opioid Overdose for 2 Data Extractors vs Emergency Medicine Physician
eFigure. Centers for Disease Control and Prevention Drug Overdose Surveillance and Epidemiology System Chief Complaint and Discharge Diagnosis Search Terms for All Suspected Opioid Overdose

This supplemental material has been provided by the authors to give readers additional information about their work.

© 2023 Griffith J et al. *JAMA Network Open.*

eTable. Interrater Reliability for Classifications of True Opioid Overdose for 2 Data Extractors vs Emergency Medicine Physician

	**Emergency medicine physician**		
	**Yes**	**No**	**Total**
**Data extractor 1**			
Yes	13	1	14
No	0	26	26
Total	13	27	40
Expected agreement		0.55	
Actual agreement		0.94	
Kappa		0.94 (95% CI = 0.84 - 1.00)	
**Data extractor 2**			
Yes	1	0	1
No	0	20	20
Total	1	20	21
Expected agreement		0.91	
Actual agreement		1.00	
Kappa		1.00 (95% CI = 1.00 - 1.00)	

Abbreviations: CI, confidence interval.

© 2023 Griffith J et al. *JAMA Network Open.*

eFigure. Centers for Disease Control and Prevention Drug Overdose Surveillance and Epidemiology System Chief Complaint and Discharge Diagnosis Search Terms for All Suspected Opioid Overdose

Variable	Automatic Inclusion	Specific terms
Inclusions		
**Discharge Diagnosis**		
ICD-9-CM poisoning	Yes	965.00, 965.01, 965.02, 965.09, E850.0, E850.1, E850.2 (also included terms with no period, e.g., “96500”)
ICD-10-CM poisoning	Yes	T40.1X1A, T40.1X4A, T40.0X1A, T40.0X4A, T40.2X1A, T40.2X4A, T40.3X1A, T40.3X4A, T40.4X1A, T40.4X4A, T40.411A, T40.414A, T40.421A, T40.424A, T40.491A, T40.494A, T40.601A, T40.604A, T40.691A, T40.694A (also included terms with no period, e.g., “T401X1A”)
ICD-10-CM opioid abuse/dependence/use with intoxication	Yes	F11.12, F11.120, F11.121, F11.122, F11.129, F11.22, F11.220, F11.221, F11.222, F11.229, F11.92, F11.920, F11.921, F11.922, F11.929 (also included terms with no period, e.g., “F1112”)
SNOWMED	Yes	295174006, 295175007, 295176008, 295165009, 242253008, 297199006, 295213004
ICD-10-CM opioid abuse/dependence use	No, must use in combination with overdose term	F11.10, F11.90, F11.20
**Chief Complaint**		
narcan or naloxone	Yes	Naloxone (narcan, evzio)
overdose term	No, must use in combination with opioid term	Poisoning (poison)<br>Overdose (overdose, overdoes, averdose, averdoes, over does, overose)<br>Nodding off<br>Snort<br>Ingestion (ingest, injest)<br>Intoxication (intoxic)<br>Unresponsive (unresponsiv)<br>Loss of consciousness (syncopy, syncope)<br>Shortness of breath (SOB), short of breath<br>Altered mental status (AMS)
opioid term	No, must use in combination	opioid, opiod, opoid, opiate, opate, opium, opium, opum, heroin, herion, heroine, HOD, speed ball,

© 2023 Griffith J et al. *JAMA Network Open.*

	with overdose term	speedball, dope, methadone, suboxone, oxyco, oxy, oxyi, percoc, vicod, fent, hydrocod, morphin, codeine, codiene, codene, oxymor, dilaud, hydromor, tramad, suboxin, buprenorphine, and other common opioid brand and generic names
Exclusions		
Chief Complaint	Exclude	Requests for drugs/drug-seeking behaviors<br>Receiving drugs for care (e.g., morphine provided for pain)<br>Detoxification<br>Withdrawal<br>Denying drug use<br>Denying loss of consciousness or shortness of breath, etc.

Reference: US Centers for Disease Control and Prevention. CDC's Drug Overdose Surveillance and Epidemiology (DOSE) System. All Opioid Overdose Case Definition. Last Reviewed December 5, 2022. Available at: https://www.cdc.gov/drugoverdose/nonfatal/case.html.

© 2023 Griffith J et al. *JAMA Network Open.*
