# Supplementary material for: Examination of the Accuracy of Existing Overdose Surveillance Systems
Source: JAMA Netw Open. 2023 Jun 28;6(6):e2320789. doi: 10.1001/jamanetworkopen.2023.20789 (PMC10308251; doi:10.1001/jamanetworkopen.2023.20789)
Supplement: Supplement 2. — Data Sharing Statement [file jamanetwopen-e2320789-s002.pdf]

Data Sharing Statement

Griffith. Examination of the Accuracy of Existing Overdose Surveillance Systems. *JAMA Netw Open*. Published June 28, 2023. doi:10.1001/jamanetworkopen.2023.20789

Data
Data available: Yes
Data types: Deidentified participant data, Data dictionary
How to access data: Data will be made available upon request by emailing study senior author Dr. Elizabeth Samuels at lizsamuels@ucla.edu.
When available: With publication

Supporting Documents
Document types: None

Additional Information
Who can access the data: Data will be made available following request approval and establishment of a data access agreement.
Types of analyses: For comparative analyses.
Mechanisms of data availability: Data will be made available with investigator support after approval of a proposal and a signed data access agreement.
